# Genetically Encoded Biosensors Based on Fluorescent Proteins

**DOI:** 10.3390/s21030795

**Published:** 2021-01-25

**Authors:** Hyunbin Kim, Jeongmin Ju, Hae Nim Lee, Hyeyeon Chun, Jihye Seong

**Affiliations:** 1 Brain Science Institute, Korea Institute of Science and Technology (KIST), Seoul 02792, Korea; binnkk@kist.re.kr (H.K.); g16512@kist.re.kr (J.J.); lhn2726@kist.re.kr (H.N.L.); hyeyoun0323@naver.com (H.C.); 2 Division of Bio-Medical Science & Technology, KIST School, Korea University of Science and Technology, Seoul 02792, Korea; 3 Department of Converging Science and Technology, Kyung Hee University, Seoul 02453, Korea

**Keywords:** fluorescent protein, genetically encoded biosensor, FRET, ddFP, BiFC, split FP, circular permutation, fluorescent timer

## Abstract

Genetically encoded biosensors based on fluorescent proteins (FPs) allow for the real-time monitoring of molecular dynamics in space and time, which are crucial for the proper functioning and regulation of complex cellular processes. Depending on the types of molecular events to be monitored, different sensing strategies need to be applied for the best design of FP-based biosensors. Here, we review genetically encoded biosensors based on FPs with various sensing strategies, for example, translocation, fluorescence resonance energy transfer (FRET), reconstitution of split FP, pH sensitivity, maturation speed, and so on. We introduce general principles of each sensing strategy and discuss critical factors to be considered if available, then provide representative examples of these FP-based biosensors. These will help in designing the best sensing strategy for the successful development of new genetically encoded biosensors based on FPs.

## 1. Introduction

After the historical discovery of green fluorescent protein (GFP) which is genetically encodable to be tagged to a protein of interest (POI) in mammalian cells [1,2], advances in the field of fluorescent protein (FP) engineering and live-cell imaging techniques have made remarkable progress in the field of cell biology [3,4]. In particular, different colors of FP have been engineered from GFP, allowing for the monitoring of multiple POIs in live cells [4]. The discovery of a red fluorescent protein dsRed [5], and the generation of mFruit progeny further expanded the color palettes of FPs [6].

In addition to fluorescent spectra, the FPs with special characteristics and different physicochemical properties have been engineered and applied to design genetically encoded biosensors for the monitoring of dynamic molecular interactions in live cells [3,7]. Here, we review the FP-based biosensors with various sensing strategies: (1) translocation of FP, (2) fluorescent resonance energy transfer (FRET), (3) dimerization-dependent FP, (4) reconstitution of split FP, (5) circularly permuted FP, (6) fluorescent timer, (7) pH-sensitive FP, and (8) photoactivatable, photoconvertible, and photoswitchable FPs.

## 2. Sensing Strategies of FP-Based Biosensors

### 2.1. Translocation of FP

**Figure 1:**
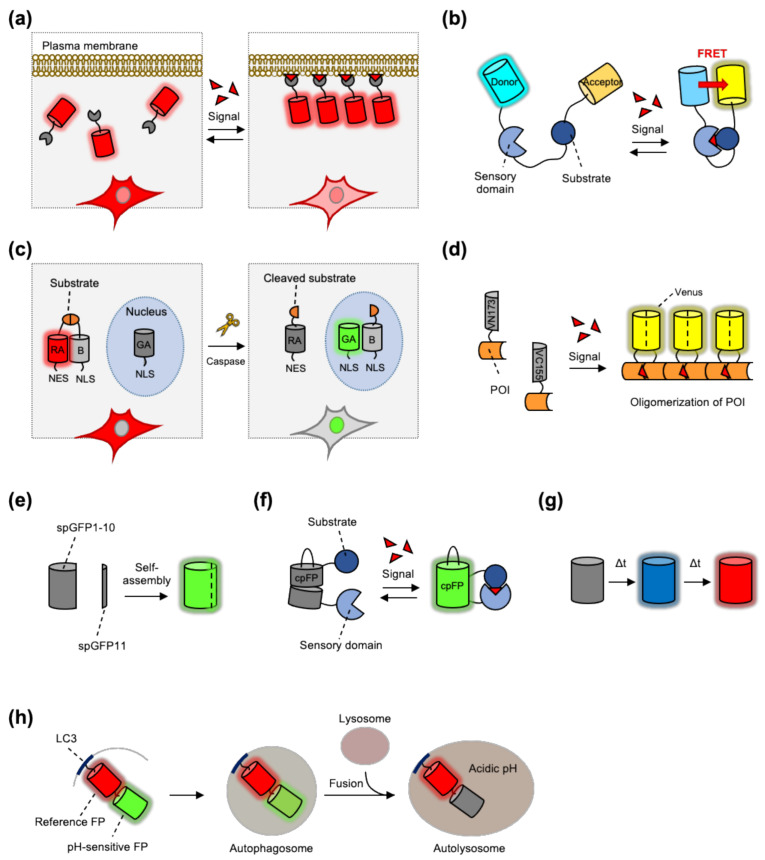
Sensing strategies of genetically encoded biosensors based on fluorescent proteins (FPs). (**a**) Example of translocation-based biosensor. The production of PtdIns at plasma membrane can be detected by the PtdIns-sensing biosensor. (**b**) Representative design of fluorescence resonance energy transfer (FRET)-based biosensor. The signal-induced conformational change of the biosensor increases the FRET between donor and acceptor FP. (**c**) Principle of ddFP-based caspase-3 biosensor. The cleavage of the substrate by caspase induces the changes of distribution (cytosol to nucleus) as well as color of fluorescence (red to green). (**d**) Principal of BiFC-based biosensor. Upon oligomerization of protein of interest (POI), BiFC fragments can be reconstituted to generate fluorescent signals. (**e**) Scheme of fluorescence reconstitution by self-assembly of split FP fragments, GFP1-10 and GFP11. (**f**) Principle of cpFP-based biosensor. The signal-induced conformation change of the biosensor increases the intensity of cpFP. (**g**) Scheme of time-dependent color change of fluorescent timer. (**h**) Design of autophagy flux sensor based on pH-sensitive FP. Autophagic vesicles become acidic during the progression of autophagy, resulting in a decrease of the intensity of the pH-sensitive FP, but not reference FP.

The first strategy for sensing dynamic molecular interactions is to monitor the translocation or distributional change of the FP-tagged protein of interest. In many signaling events, the signaling molecules translocate to different subcellular regions to be functional or interact with downstream signaling molecules [8]. Thus, visualizing the location of the POIs or the protein that specifically binds to the POIs can be used to report on the particular molecular events in live cells (Figure 1a).

When we design a genetically encoded sensor for this strategy, it is important to make sure that the fusion of the FP does not disturb the original localization or function of the attached POIs. For example, when we monitor focal adhesion dynamics by FP-tagged paxillin, a FP should be fused to the N-terminal of the paxillin because its targeting motif, i.e., four LIM domains, is located at the C-terminal [9].

In addition, to detect the distribution of POIs accurately, the fused FP should be monomeric [10]. The original GFP is a weak dimer [1] and dsRed is a tetramer [11]. If the POI is fused to a dimeric or tetrameric FP, the FP-tagged POI may be mislocalized or aggregated due to the oligomerizing tendency of the FP itself [10]. Continuous efforts to engineer monomeric FPs are being made such as A206K mutation of GFP [12], thus various colors of monomeric FPs are currently available [3,13].

As representative examples, phosphoinositides (PtdIns)-sensing biosensors have been designed to detect their translocation or distributional change [14] (Figure 1a). PtdIns are lipid-signaling molecules that play crucial roles in membrane trafficking and diverse cell signaling pathways [15]. Depending on the site and number of phosphate groups attached to the inositol ring, PtdIns can be classified into seven different forms, i.e., PtdIns3P, PtdIns4P, PtdIns5P, PtdIns(3,4)P_2_, PtdIns(3,5)P_2_, PtdIns(4,5)P_2,_ and PtdIns(3,4,5)P_3_. Each form interacts with particular downstream molecules via its PtdIns binding domains, such as the PH, FYVE, PX, and PDZ domains [16]. Thus, the PtdIns biosensors are designed to comprise the PtdIns binding domain tagged with a monomeric FP. For example, the PtdIns(4,5)P_2_ sensor contains a PH domain from PLC-δ1 and a monomeric GFP [17]. In addition, the FP-tagged PH domain from Akt or Btk has been used to monitor the translocation of PtdIns(3,4,5)P_3_ [18,19]. Likewise, the FP-tagged PX domain of the p40phox protein and the tandem of FYVE domains from EEA1 or Hrs proteins have been used as biosensors detecting the distribution of PI(3)P [20,21].

These PtdIns sensors can report the real-time activities of their metabolizing enzymes, e.g., kinases and phosphatases. For example, when phosphoinositide 3-kinase (PI3K) is activated at the plasma membrane, it specifically phosphorylates PtdIns(4,5)P_2_ generating PtdIns(3,4,5)P_3_. Thus, the distributional change of PtdIns(3,4,5)P_3_ from the cytosol to the plasma membrane reports the real-time activity of PI3K. In addition, when PtdIns(3,4,5)P_3_ is dephosphorylated by a phosphatase SHIP2, PtdIns(3,4)P_2_ is generated which can initiate endocytosis [22]. Thus, a PtdIns(3,4)P_2_ sensor consisting of the PH domain from TAPP1 [23] can be applied to monitor the SHIP2 activity in live cells [24]. Tandem PH domains from TAPP1 can enhance the sensitivity of the translocation-based PtdIns(3,4)P_2_ biosensor [25].

Another interesting example of a translocation-based FP biosensor is the conformation sensor for the G-protein coupled receptor (GPCR). For example, an active conformation of the beta-2 adrenergic receptor (B2AR), a prototypical GPCR, can be specifically captured by a nanobody-based sensor Nb80-GFP [26]. The Nb80-GFP is composed of a recombinant single-domain antibody from camelid that specifically binds to the intracellular pocket of the active state of B2AR at the plasma membrane [27]. Thus, in response to agonist treatment, the translocation of cytosolic Nb80-GFP to the plasma membrane indicates the active conformation of B2AR. Nanobodies can be synthetically created with excellent antibody specificity; thus, these nanobody-based FP sensors can be further applied to visualize various molecular dynamics.

### 2.2. Fluorescent Resonance Energy Transfer

Fluorescent resonance energy transfer (FRET) is a physical phenomenon of energy transfer between two chromophores with overlapping spectra in proximity (<10 nm) [28]. The signaling pathways during various cellular processes are dependent on the proximal interactions between signaling molecules, thus, FRET-based biosensors have been widely utilized for the monitoring of these molecular interactions [29,30] (Figure 1b).

For efficient FRET between the donor and acceptor FPs, the emission spectrum of the donor FP should overlap with the excitation spectrum of the acceptor FP [31]. The distance between donor and acceptor FPs and their relative orientation should be also considered. In addition, several parameters, such as the quantum yield (QY) of the donor and the extinction coefficient (EC) of the acceptor can contribute to the efficiency of FRET [32].

The best-known FRET pair in FRET-based biosensors is cyan fluorescent protein (CFP) and yellow fluorescent protein (YFP) due to its overlapping spectrum. However, the CFP–YFP pair have some limitations, for example cross-talk between their excitation and emission spectra [33], the low QY of CFP, and the relatively poor photostability and pH-stability of YFP [34,35]. After engineering efforts to improve these issues, a CFP variant (mTurquoise2) with relatively high QY (0.93) [35] and a bright green–yellow FP (mNeongreen) with improved stability have been shown to be a great FRET pair [36]. 

The green–red FRET pair displays greater spectral separation and less phototoxicity [29]. However, red FPs are less bright and their property of forming tetramers interferes with the proper measurement of FRET, thus monomeric red FPs with enhanced brightness and photostability have been further engineered for the better FRET [37].

In addition, FPs with a large Stokes shift (LSS), which display a large gap between the excitation and emission spectra [38], are useful to apply dual FRET imaging for the investigation of multiple molecular events in live cells. For example, the combination of LSSmOrange-mKate2 and CFP–YFP pairs was successfully applied for the simultaneous monitoring of caspase-3 activity and intracellular Ca^2+^ level [39]. In that experiment, both donors, CFP and LSSmOrange, were excited at 440 nm, and the resulting FRET to the acceptors could be detected at 530 nm for YFP and 630 nm for mKate2, thus allowing dual FRET imaging of two different biosensors.

**Figure 2:**
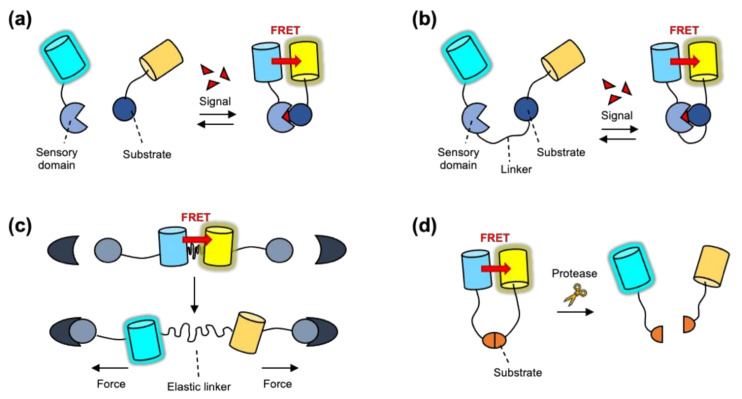
Designs of FRET biosensors (**a**) Representative design of an intermolecular FRET biosensor. The signal-induced interaction of the modified substrate and the sensory domain results in the increase of FRET between donor and acceptor FP. (**b**) Representative design of an intramolecular FRET biosensor. The signal-induced conformational change of the biosensor increases the FRET level. (**c**) Design of a FRET-based tension sensor. Its FRET level is designed to decrease by the applied tensional force. (**d**) Design of a FRET-based protease sensor. The activated protease cleaves its substrate, resulting in the decreased FRET of the biosensor.

To measure protein–protein interactions by the FRET biosensors, one POI is tagged by a donor FP and the other by an acceptor FP, thus the FRET signals can be detected when these POIs physically interact with each other in proximity. This simple strategy can be applied to intermolecular or intramolecular FRET sensors [29,40] (Figure 2a,b). Intermolecular FRET-based biosensors can be applied to detect protein–protein interaction or molecular proximity [41,42] (Figure 2a). However, in the case of intermolecular FRET sensor, the donor and acceptor FP-containing parts may display different expression levels and/or distribution at different subcellular regions. In addition, each part may be able to interact with endogenous proteins. These limitations interfere with the accurate measurement of the FRET. 

In contrast, intramolecular FRET sensors contain both the acceptor and donor FPs, thus their expression and distribution can be equally controlled. Various designs of intramolecular FRET biosensors can be developed depending on the molecular events to be monitored. The first design is composed of the specific substrate for target signaling molecule and the sensory domain between a FRET pair (Figure 2b). As a representative example, the FRET-based kinase biosensor contains the specific substrate sequence, which can be phosphorylated by the target kinase, and the sensory domain that binds to the phosphorylated substrate such as SH2 domain resulting in the changes of FRET [43,44]. In addition, FRET-based biosensors detecting the activation of small Rho GTPase include the Rho GTPase itself as a substrate of the biosensor. When Rho GTPase is activated, its bound GDP is exchanged to GTP, thus, it can subsequently bind to the sensory domain from its downstream molecule, resulting in the changes of FRET [45,46].

Another design of intramolecular FRET biosensor is a FRET-based tension sensor which includes a tension sensing module consisting of donor and acceptor FPs connected by an elastic linker [47] (Figure 2c). This tension sensing module is further attached to the head and tail of vinculin, which is a key molecule to connect the focal adhesions to actin cytoskeleton [48]. Thus, the tensional force at focal adhesions extends the elastic linker in this sensor causing the decrease in FRET, allowing for the visualization of cellular tension in live cells.

The other design of intramolecular FRET biosensor is a protease sensor consisting of specific substrate for a target protease between a FRET pair (Figure 2d). The strong FRET between the FRET pair will be decreased when the activated proteases cleave the substrate in the biosensor and thus separate the donor and acceptor FPs. Specific protease sensors have been developed based on particular substrate sequences, for example caspase-3/7 (DEVD; Asp-Glu-Val-Asp) [49], caspase-8 (IETD; Ile-Glu-Thr-Asp) [50], and caspase-9 (LEHD; Leu-Glu-His-Asp) [51]. However, this protease-mediated cleavage of substrate sequence is irreversible reaction, thus the FRET measurement with these FRET biosensors is not reversible.

### 2.3. Dimerization-Dependent FP

Dimerization-dependent fluorescent proteins (ddFPs) can be an alternative method for sensing protein–protein interactions. Similar to FRET-based biosensors, a ddFP system is composed of two copies of FPs as a pair [52]. One copy in the ddFP pair contains a chromophore (copy-A), while the other copy does not (copy-B). When they exist separately, the fluorescence of copy-A is dim; however, the ddFP system becomes bright when copy-A and copy-B form heterodimers. Thus, we can detect protein–protein interactions by measuring the increase in the fluorescence brightness of the ddFP system (Figure 1c).

The first ddFP system was red ddFP, which was developed using a red FP, dTomato [52]. Green and yellow ddFP were followed, expanding the color palette of the systems [53]. As the copy-A in different colors of ddFPs competes to bind the same copy-B, a fluorescent protein exchange (FPX) strategy can be applied to design the biosensors, which show protein activity in terms of color changes. For example, the intermolecular caspase-3 FPX biosensor is composed of two parts. The first part contains RA (red copy-A)-NES and copy-B-NLS, connected with the caspase-3 substrate sequence DEVD; the second part is GA (green copy-A)-NLS [54] (Figure 1c). In the default state, the heterodimer of RA and the copy-B display red fluorescence in the cytosol. Upon caspase-3 activation, the substrate DEVD between RA and B is cleaved and the released B-NLS can translocate into the nucleus, where it binds to GA-NLS and displays green fluorescence. Therefore, the activity of caspase-3 can be monitored by the FPX-mediated changes of fluorescent colors at subcellular locations.

In addition, the intramolecular caspase-3 FPX biosensor is composed of RA, copy-B, caspase-3 substrate DEVD, and GA [54]. In this conformation, the copy-B is designed to bind to GA, displaying a green color, while the activation of the caspase-3 cleaves the DEVD sequence changing its color from green to red. Similarly, the intramolecular Ca^2+^ FPX biosensor is designed to be RA-CaM-B-M13-GA, which can report the Ca^2+^ levels by monitoring the red/green fluorescence ratio [54].

More recently, the ddFP-based biosensors for small GTPases have been introduced [55]. These intermolecular biosensors are composed of two parts; the first part is the GTPase itself attached to GA, and the second part is the copy-B-tagged the RBD domain from its corresponding effector. For example, the activation of KRas can be visualized by the ddFP-based G-KRas system which is composed of GA-KRas and copy-B-RBD_Raf1_. When the activated KRas binds to the RBD domain from its effector Raf1, the heterodimer between GA and copy-B can be formed to increase the brightness of green fluorescence, representing the active state of KRas in live cells. These ddFP-based biosensors for small GTPases were successfully utilized to monitor the spatiotemporal activity of the small GTPases in the single dendritic spines and brains of freely behaving mice [55].

Unlike the FRET systems, each ddFP system uses only one hue, thus, the green ddFP-based biosensors can be applied together with red-shifted sensors, allowing the simultaneous monitoring of multiplex signaling events in the same cell. In addition, the red ddFP system can be combined with green sensors or blue-light sensitive optobiochemical tools [56]. Expanding color palettes of the ddFP system will be further beneficial to study complex molecular dynamics in live cells.

Due to different affinities of GA and RA to the copy B [54], one needs to be careful to apply multicolor ddFP systems to quantitative assays. In addition to the engineering efforts for the copy B [55], further engineering on XA (X for different colors) will improve the multicolor ddFP systems.

### 2.4. Reconstitution of Split FP

The reconstitution of split fluorescent proteins by the fusion of its non-fluorescent fragments has been applied to detect the protein–protein interactions [57] (Figure 1d). The chromophore of GFP is protected by a beta barrel structure composed of 11 beta-strands [58]. Splitting a FP into two fragments, 7 and 4 strands each, results in the loss of fluorescence, but interestingly, when these two fragments are in proximal they can reconstitute and recover its fluorescent property [59]. This is called bimolecular fluorescence complementation (BiFC), and by fusing the POIs to each fragment, we can monitor the protein–protein interactions or aggregations by detecting the increased fluorescence [60]. The most widely used BiFC system was developed by splitting Venus, an improved yellow FP, into N-terminal 1–158 amino acids (VN158) and C-terminal 159–239 residues (VN159) [61], or VN173–VN155, which showed higher complementation efficiency and signal to noise ratio [62]. The color palette has been further broadened by the BiFC systems from a cyan FP Cerulean [62], and from red FPs such as mRFP, mCherry, and mKate [63,64,65].

The BiFC-based biosensor was first applied to detect the interaction between the basic region leucine zipper (bZIP) domains of Fos and Jun in live cells [61]. The hypothetical t_1/2_ of bZIP association was less than a second while for BiFC formation and fluorescence maturation it was 50 min, indicating that the BiFC occurs subsequent to the association of the attached bZIP domains. The BiFC principle has also been applied to detect protein aggregation in neurodegenerative diseases. For example, both N- and C-terminal fragments of Venus were fused to alpha-synuclein [66], which aggregates to cause Lewy pathology in Parkinson’s disease (PD) [67,68]. This BiFC-based alpha-synuclein sensor can be applied to visualize the cell-to-cell transmission of alpha-synuclein during the progression of PD [66,69]. In addition, tau aggregation, a pathological hallmark of Alzheimer’s disease (AD), was successfully detected using a BiFC-based biosensor in live cells [70]. Both fragments of Venus were fused to full length tau, allowing for the detection of tau aggregation by BiFC fluorescence in live cells (Figure 1d). Transgenic mice expressing mutant human Tau P301L-BiFC were recently generated to monitor pathological tau oligomerization in AD [71].

Interestingly, it has been shown that the BiFC system and FRET technique can be combined [72]. In this design, Cerulean and the Venus-based BiFC system were used as the donor and acceptor FPs for cyan–yellow FRET. This system was applied to visualize a ternary complex of Fos-Jun-nuclear factor of activated T cells (NFAT) [73]. The BiFC fragments of Venus were fused to bJun and bFos, and Cerulean was fused to NFAT. The dimerization of bJun and bFos reconstituted the BiFC fragments of Venus. When NFAT interacts with this bJun-bFos complex, the FRET between Cerulean and Venus can be observed. Thus, the BiFC-FRET system allows the validation of protein interactions and the monitoring of spatial information of three proteins in live cells.

Furthermore, the BiFC system from large Stokes shift-FP was developed from CyOFP1 (CyOFP1-N151 and CyOFP-C152) and mT-Sapphire (mT-Spphire-N154 and mT-Spphire-C155) [74]. Utilizing the BiFC systems from mT-Sapphire and Cerulean, two distinct protein–protein interactions can be monitored by a single violet excitation and two emission wavelengths, cyan for Cerulean and green for mT-Sapphire. Similarly, cyan light can be used to excite two BiFC systems from Venus and CyOFP1. These multicolor BiFC systems allow the simultaneous monitoring of multiple protein interactions in live cells.

The BiFC-based biosensor is based on the irreversible association of non-fluorescent fragments, subsequent protein folding and chromophore maturation. Thus, it is limited to investigate the temporal information of protein dynamics. In addition, to accurately detect the interactions of POIs attached to the BiFC system, it is important to minimize the nonspecific self-assembly of the FP fragments to avoid a false-positive signal.

Interestingly, this limitation of self-assembly was alternatively applied to measure the proximity of two membranes or organelles tagged with each FP fragment [75]. The self-assembling FP fragments were generated by splitting the 1–10 strands (spGFP1-10) and 11th strand (spGFP11) of superfolder GFP (sfGFP) [76] (Figure 1e). These two fragments were separately fused to pre- or post-synaptic membranes of neurons, thus, when synapse is formed, these FP fragments can be assembled to reconstitute the green fluorescence. This technique, called GFP reconstitution across synaptic partners (GRASP), can thus visualize high-resolution anatomy of synaptic cleft [77,78]. More recently, spGFP11 is fused to synaptic vesicle protein synaptobrevin and spGFP1-10 is tethered at post-synaptic membrane, thus the neuronal activity-induced release of synaptic vesicles can be visualized by this activity-dependent GRASP technique [79].

In addition to green split FP for self-assembly, different colors of split FPs have been developed. For example, red color of split FP system was engineered from superfolder Cherry [80]. Cyan and yellow split FPs were developed by introducing key mutations on spXFP1-10 (X for cyan or yellow), which were applied for multi-color labelling of active synapses [79]. More recently, the improved multi-color GRASP systems were successfully applied to identify the specific neuronal sites for memory storage [81].

### 2.5. Circularly Permutated FP

Because the N- and C-termini of a fluorescent protein are located in the same direction, they can be connected with a linker creating new termini near the chromophore [82]. In this circularly permuted FP (cpFP), the new termini can be further fused to the sensing domains whose conformational rearrangement can modulate the fluorescent intensity of cpFP (Figure 1f). Compared with the original FP, the cpFP displays lower fluorescence intensity due to its relatively weak folding near chromophore, thus the conformational rearrangement of the sensing domains can enhance the brightness of cpFP.

Various biosensors have been developed based on cpFP [83]. For example, the first cpFP-based biosensors, GCaMP and pericam [84,85], were developed to detect the intracellular Ca^2+^ levels. These Ca^2+^ sensors are composed of cpGFP fused to calmodulin and M13 in each new end. When Ca^2+^ binds to calmodulin, its subsequent interaction with M13 causes the conformational rearrangement, resulting in the increased brightness of the cpGFP (Figure 1f). Different colors of cpFP-based Ca^2+^ sensors have been also developed for the simultaneous monitoring of multiple signals. For example, red Ca^2+^ sensors R-GECO [86,87] and RCaMP [87,88] were developed from cp-mApple and cp-mRuby, respectively. The Ca^2+^ sensors were continuously improved; thus, we can currently monitor dynamic changes of intracellular Ca^2+^ levels in living animal as well as live cells [89,90,91,92,93].

In addition to Ca^2+^ sensors, cpFP-based sensors to detect cofactors [94], cAMP [95], ATP [96,97], or neurotransmitters such as glutamate and GABA [98,99] were developed by inserting specific sensing domains to cp-FP. Different from these cytosolic cpFP-based sensors detecting diffusible signaling molecules, the cpFP-based voltage sensor, named ASAP, was designed by fusion of cpFP module to voltage-sensing domains tethered at plasma-membrane [100,101]. The changes in membrane potential induces the conformational change of voltage-sensing domains, thus resulting in the increased brightness of the cpFP. By rational design and structure-based mutagenesis, the cpFP-based voltage sensors can visualize fast kinetics of membrane potential in living neurons and animals [102].

More recently, cpFP-based sensors for metabotropic neurotransmitter receptors have been reported. These receptors are types of GPCRs; thus, in response to binding to neurotransmitters, the conformational changes of the receptor initiate intracellular signaling related to G proteins [103]. The first cpFP-based dopamine receptor sensors were developed by inserting the cpGFP module into the intracellular loop 3 (ICL3) of dopamine receptors [104,105]. When the dopamine binds to the receptor, the subsequent conformational change of ICL3 regions causes the increased brightness of the inserted cpGFP.

This strategy has been further applied to develop other neurotransmitter receptor sensors. For example, cp-based sensors detecting the activity of the receptors for acetylcholine [106], norepinephrine [107], adenosine [108], and serotonin [109] were developed, allowing the monitoring of spatiotemporal activity of various neurotransmitter receptors. In addition to green sensors, red dopamine receptor sensors have recently been developed utilizing cp-mApple [110,111], and thereby different neurotransmitter signals can be simultaneously monitored in living neurons and animals.

### 2.6. Fluorescent Timer

The first fluorescent timer (FT) was developed from DsRed-E5, a DsRed mutant with two substitutions V105A and S197T [112]. In particular, S197T is suggested to directly contact the chromophore as an analogue of T203 in GFP, enabling the mutant E5 to exhibit a green intermediate fluorescence before its full maturation for red fluorescence. The green-to-red color conversion of DsRed-E5 is time-dependent, thus this special FP can be utilized as a fluorescent timer to sense the relative ages of the attached POIs (Figure 1g). 

**Figure 3:**
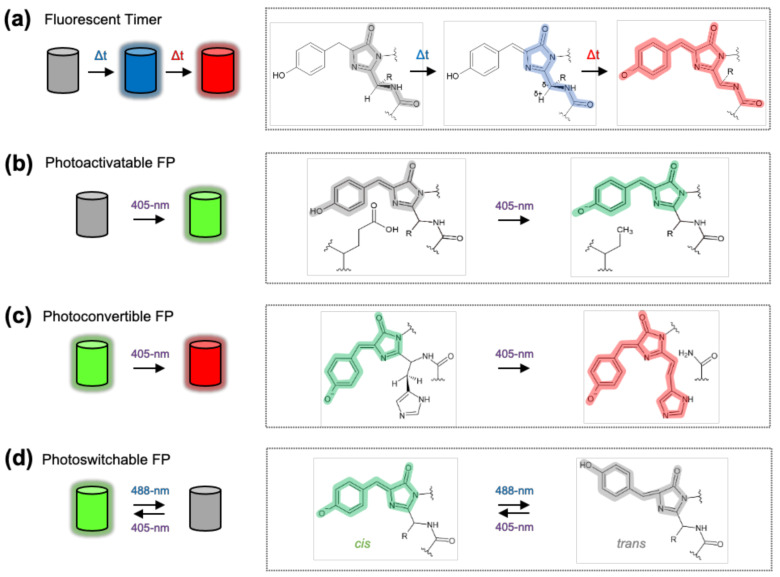
Mechanism of fluorescent timer, photoactivation, photoconversion and photoswitching. (**a**) Scheme of monomeric fluorescent timer (FT) and its chromophore structure (box). Slow maturation process (oxidation) of chromophore of FT allows the alteration of emission wavelength alteration over time. (**b**) Scheme of photoactivatable GFP (PA-GFP) and its chromophore structure (box). Illumination of 405-nm light induces the decarboxylation of the key residue near the chromophore of PA-GFP, transforming the chromophore to be a fluorescent emitting form. (**c**) Scheme of photoconvertible FP (EosFP) and its chromophore structure (box). Illumination of 405-nm light induces the cleavage of peptide backbone near chromophore, changing the color of emitting fluorescence from green to red. (**d**) Scheme of photoswitchable FP (Dronpa) and its chromophore structure (box). Illumination of 488-nm light induces cis-to-trans isomerization of chromophore turning off the fluorescence, while 405-nm light reverses it by trans-to-cis isomerization turning on the fluorescence. Highlighting blue, green, red colors on the chromophores display the color of emitting fluorescence, and gray represent non-fluorescent state of chromophore.

DsRed-E5 is a tetramer that may prevent the proper tagging of target proteins. Thus, monomeric FTs were further engineered from mCherry with key mutations on K69R, L84W, and M18V/L [113]. These monomeric FTs show a blue-to-red conversion over time during chromophore maturation process (Figure 3a). Additional mutations on A224S or A179V influence the rates for chromophore maturation, thus fast-FT, medium-FT, and slow-FT can be developed. They show the maxima of blue fluorescence at 0.25, 1.2, and 9.8 h, and the half-maxima of red fluorescence at 7.1, 3.9, and 28 h, respectively. These different speeds of FTs can be applied to sense the ages of the target proteins with various time scales. For example, medium-FT was selected to detect the relative ages of the lysosome-associated membrane protein type 2A (LAMP2A) at endosomal compartments.

Another color of monomeric FT, mK-GO, was engineered from a monomeric version of Kusabira Orange (mKO) by introducing six mutations (K49E, P70V, K185E, K188E, S192D, and S196G) [114]. The mK-GO changes its color from green to orange during the chromophore maturation process. The orange/green ratio increases over time and reaches a plateau at around 10 h. For example, age-dependent vesicle exocytosis was investigated utilizing mK-GO-tagged neuropeptide Y or a tissue-type plasminogen activator.

In addition to single FTs, tandem FTs (tFTs) were reported which utilizes the combination of two FPs with different maturation kinetics as well as separate spectral profiles [115]. In the study, sfGFP that rapidly matures within several minutes and mCherry with a maturation half-time of 40 min, were combined as a tFT. The mCherry-sfGFP timer can measure the ages of the fused POIs from red-to-green fluorescence ratios. Therefore, fluorescent timers can be applied to develop biosensors to monitor protein turnover or mobility in live cells.

### 2.7. pH-Sensitive FP

The excitation spectrum of wild-type GFP is bimodal with two peaks at 395 and 475 nm, which are suggested as the protonated and deprotonated states at Tyr 66 of the chromophore [116]. This contrast of peaks in the excitation spectrum upon pH changes can be further increased by the mutations on the residues near the protein-relay network of Tyr 66 (S202H, E132D, S147E, N149L, N164I, K166Q, I167V, R168H, and L220F) [117]. This ratiometic pH-sensitive FP was named pHluorin, which shows a reversible ratio change of the excitation peaks in the physiological range of pH between 5.5 and 7.

Another version of ecliptic pHluorin (S147D, N149Q, T161I, S202F, Q204T, and A206T), in contrast, shows a gradual decrease in the excitation peak at 475 nm, and it becomes eclipsed at a pH below 6 [117]. The ecliptic pHluorin was further engineered to increase its brightness by introducing additional mutations from EGFP (F64L and S65T), and this superecliptic pHluorin (SEP) shows a 50-fold change of fluorescent intensity in the range of physiological pH [118].

A red fluorescent pH-sensitive FP, pHTomato, was engineered from mRFP and mStrawberry [119]. pHTomato was fused to a vesicular membrane protein synaptophysin to generate sypHTomato and was applied for the monitoring of synaptic vesicle exocytosis together with a green GCaMP sensor. The fluorescence fold-change of pH-sensitive red FP was further improved in the orange pHoran4 (17-fold) and red pHuji (22-fold), which were engineered from mOrange and mApple, respectively [120].

The pH-sensitive FP can be applied to monitor the status of various cellular processes. For example, the exocytosis or recycling of synaptic vesicles has been visualized by tagging the pH-sensitive FPs to synaptophysin or VAMP [117,119]. While acidic pH inside the synaptic vesicles results in the fluorescence quenching of the pH-sensitive FPs, it becomes bright when exposed to the neutral extracellular environment after fusion to the plasma membrane [121], thus allowing the detection of exocytosis or recycling of synaptic vesicles in neurons. In addition to mammalian cells, the pH-sensitive FP-based biosensors have been applied to investigate the intra- and extracellular pH changes in plant cells [122,123].

Another representative application of pH-sensing FPs is the design of an autophagy progression sensor, by tagging pH-sensitive FP to the LC3 which is tethered in the autophagosome [124,125,126]. During the progression of autophagy, the autophagosome is fused to the lysosome, and the inside pH of this autolysosome becomes acidic for the degradation of the cargo proteins. Therefore, the autophagy stages can be distinguished by pH-sensitive FPs [127]. For example, in the mRFP-GFP-tagged LC3, GFP loses its fluorescence in the acidic pH, but mRFP does not, thus we can speculate the pH inside the autophagic vesicles by measuring the green/red ratio (Figure 1h).

Recently, an autophagy progression sensor was further improved to more specifically identify each stage of autophagy [128]. In this red–green–blue FP tagged LC3 (RGB-LC3), mTagBFP (pK_a_ = 2.7), mApple (pK_a_ = 6.5), and SEP (pK_a_ = 7.2) were chosen as a blue reference FP, a pH-sensitive red FP, and a highly pH-sensitive green FP. Thus, by measuring the green/blue and the red/blue signal ratios, RGB-LC3 allows for more accurate calculation of the pH inside the autophagic vesicles during autophagy progression.

Interestingly, a pH-stable cyan FP (mTurquoise2) and a pH-sensitive yellow FP (EYFP) were combined to generate pH-Lemon, which could report the pH by detection of FRET between mTurquoise2 and EYFP [129]. As the FRET will decrease in the acidic environment due to the quenching of EYFP, this FRET pair was tagged with LC3 to report the progression of autophagy.

### 2.8. Photoactivatable, Photoconvertible, and Photoswitchable FP

Upon light stimulation, some FPs become fluorescent from a non-fluorescent state (photoactivatable) [130], change the colors (photoconvertible) [131] or reversibly switch on and off (photoswitchable) [132] by the light-induced rearrangement or isomerization of the chromophore [133] (Figure 3). The unique features of these special FPs can be further combined to previously described sensing techniques.

Photoactivatable FP, such as PA-GFP, increases its green fluorescence 100 times by illumination of blue light (Figure 3b) [130]. This special feature of PA-FP can be applied to investigate intracellular protein dynamics. For example, the PA-GFP fused to a lysosomal protein LAMP-2 was activated at the nucleus by local illumination, the rate of interlysosomal membrane exchange can be investigated by tracking the photoactivated FP-fused LAMP-2 [130]. Furthermore, the low background signal and controllable fluorescent intensity of PA-FP were applied to develop a super resolution imaging technique, called fluorescence photoactivation-localization microscopy (FPALM) [134], which provides accurate information of POI’s distribution at ultra-high resolution.

Photoconvertible FP, such as mEos, changes its color from green to red upon blue illumination (Figure 3c) [131]. It can function as a highlighter to track the attached POIs and related cellular dynamics [135]. More interestingly, photoconvertible FPs can be further combined with the cpFP technique to create a highlightable Ca^2+^ sensor, CaMPARI [136]. CaMPARI is composed of cp-mEos and the Ca^2+^ sensing modules, CaM and M13, from GCaMP. When Ca^2+^ binds to the sensing modules in CaMPARI, the cp-mEos becomes fluorescent green. This green state can be converted to a red state, if the subsequent illumination with blue light is applied. Thus, CaMPARI can capture active neuronal population with the elevated Ca^2+^ level by converting its color to red with illumination during particular behavior in live animals. The CaMPARI was further improved to generate CaMPARI2 by reducing the basal photoconversion, increasing the Ca^2+^ exchange kinetics and fluorescence after chemical fixation [137].

Photoswitchable FP can be fluorescent on and off, reversibly, upon the illumination of specific wavelengths of light (Figure 3d). For example, Dronpa which can be fluorescent on by 405-nm light and off by 488-nm light [132]. This photoswitchable FP has been applied to generate a special BiFC system named as reconstituted fluorescence-based stochastic optical fluctuation imaging (refSOFI) [138]. In this system, the POI1 and POI2, for example STIM1 and ORAI1, can be attached to each fragment of Dronpa. Thus, the protein–protein interaction can result in the green signal of Dronpa, which then further switched on and off for super-resolution imaging. Thus, the combination of photoswitchable FP and BiFC techniques allows the investigation of spatial information of protein interactions at a super-resolution scale.

## 3. Conclusions

In this review, we have discussed various strategies of genetically encoded biosensors based on different physicochemical properties and special features of fluorescent proteins, for example FRET, ddFP, the reconstitution of split FP, circular permutation, pH sensitivity, maturation speed, and photoactivation/photoconversion/photoswitching. Depending on the target proteins or molecular events to be monitored, different strategies need to be carefully chosen for the development of successful biosensors. Interestingly, combination of these techniques, for example BiFC-FRET, pH sensitive FP-FRET, photoconvertible FP-cpFP and photoswitchable FP-BiFC, allowed the development of interesting biosensors for unique purposes. There are surely further possible combinations for novel genetically encoded biosensors that will uncover physiologically important, but not yet visualized, dynamic molecular signals in living cells and animals. These advances in FP-based biosensors will allow for the discovery of the underlying scientific mechanisms of dynamic and complex cellular processes.

## Data Availability

Data sharing is not applicable to this article.

## Abbreviations

FP	Fluorescent protein
GFP	Green fluorescent protein
POI	Protein of interest
FRET	Fluorescent resonance energy transfer
LIM	LIN-11, Isl-1 and MEC-3
RFP	Red fluorescent protein
PtdIns	Phosphoinositide
PH	Pleckstrin homology
FYVE	Fab1p, YOTB, Vps27p and EEA1
PX	Phox homology
PDZ	PSD95, DLG1 and ZO1
PLC-δ1	Phospholipase C-δ1
EEA	Early endosome antigen 1
Hrs	Hepatocyte growth factor-regulated tyrosine kinase substrate
PI3K	Phosphoinositide 3-kinase
SHIP2	Src homology domain containing inositol 5-phosphatase
TAPP1	Tandem-PH-domain-containing protein 1
GPCR	G-protein coupled receptor
B2AR	Beta-2 adrenergic receptor
Nb80	Nanobody 80
QY	Quantum yield
EC	Extinction coefficient
CFP	Cyan fluorescent protein
YFP	Yellow fluorescent protein
LSS	Large Stokes shift
SH2	Src homology 2
GDP	Guanosine diphosphate
GTP	Guanosine triphosphate
ddFP	Dimerization-dependent fluorescent protein
FPX	Fluorescent protein exchange
RA	Red copy-A
NES	Nuclear export signal
NLS	Nuclear localization signal
GA	Green copy-A
CaM	Calmodulin
RBD	Ras-binding domain
CRIB	Cdc42/Rac1 interactive binding
BiFC	Bimolecular fluorescence complementation
bZIP	Basic leucine zipper
PD	Parkinson’s disease
AD	Alzheimer’s disease
NFAT	Nuclear factor of activated T cells
spGFP	Split GFP
sfGFP	Superfolder GFP
GRASP	GFP reconstitution across synaptic partners
cpFP	Circularly permuted FP
cAMP	Cyclic adenosine monophosphate
ASAP	Accelerated Sensor of Action Potentials
ICL3	Intracellular loop 3
FT	Fluorescent timer
LAMP2A	Lysosome-associated membrane protein type 2A
mK-GO	Monomeric Kusabira Green Orange
mKO	Monomeric Kusabira Orange
tFT	Tandem FT
SEP	Superecliptic pHluorin
VAMP	Vesicle-associated membrane protein
LC3	Microtubule-associated protein 1A/1B-light chain 3
EYFP	Enhanced yellow fluorescent protein
PA	Photoactivatable
mEos	Monomeric Eos probes
CaMPARI	Calcium Modulated Photoactivatable Ratiometric Integrator
refSOFI	Reconstituted fluorescence-based stochastic optical fluctuation imaging
STIM1	Stromal interaction molecule 1
ORAI1	Calcium release-activated calcium channel protein 1
